# Haptic Glove Systems in Combination with Semi-Immersive Virtual Reality for Upper Extremity Motor Rehabilitation after Stroke: A Systematic Review and Meta-Analysis

**DOI:** 10.3390/ijerph191610378

**Published:** 2022-08-20

**Authors:** Diego Fernández-Vázquez, Roberto Cano-de-la-Cuerda, Víctor Navarro-López

**Affiliations:** Department of Physical Therapy, Occupational Therapy, Rehabilitation and Physical Medicine, Faculty of Health Sciences, Universidad Rey Juan Carlos, 28922 Madrid, Spain

**Keywords:** haptic glove, virtual reality, stroke, upper extremity, motor function

## Abstract

Background: The effectiveness of the virtual reality (VR) for the upper extremity (UE) motor rehabilitation after stroke has been widely studied. However, the effectiveness of the combination between rehabilitation gloves and semi-immersive VR (SVR) compared to conventional treatment has not yet been studied. Methods: A systematic search was conducted in Pubmed, Web of Science, PEDRo, and Scopus, Cochrane, CINHAAL databases from inception to May 2022. Randomized controlled trials were included if patients were under rehabilitation with haptic gloves combined with SVR intervention focused on the UE rehabilitation in stroke patients. Risk of bias and methodological quality were evaluated with the Physiotherapy Evidence Database (PEDro), and the modified Cochrane library criteria. A random effects model was used for the quantitative assessment of the included studies using the standard mean difference with a 95% confidence interval. Heterogeneity among the included studies was assessed using Cochran’s Q test and the incoherence index (I^2^). Results: After a first screening, seven studies were included. Significant differences with a 95% confidence interval were obtained in favor of the rehabilitation glove combined with SVR in the short term (SMD—standardized mean differences = 0.38, 95% CI—confidence interval = 0.20; 0.56; Z: 4.24; *p* =< 0.001). In the long term, only the studies that performed an intervention based in rehabilitation glove combined with SVR with also included rehabilitation were able to maintain the improvements (SMD = 0.71, 95% CI = 0.40; 1.02; Z: 4.48; *p* =< 0.001). Conclusions: The combined use of rehabilitation haptic gloves and SVR with conventional rehabilitation produces significant improvements with respect to conventional rehabilitation treatment alone in terms of functionality of the UE in stroke patients.

## 1. Introduction

Stroke affects around 15 million people worldwide per year, with a mortality rate of about 30% in the first year, being the most prevalent and the first leading cause of long-term disability disease in the world [1]. After a stroke, people generally suffer movement impairments that limit them in different tasks such as self-care, writing, work activities or driving.

Impaired hand function is one of the most common effects of stroke, which refers to the ability to grasp and manipulate objects, closely related to independence [2]. In this framework, Virtual Reality (VR) emerges as a high interesting tool in the treatment of the upper extremity (UE) impairments in stroke patients. VR is defined as a computer system used to create a virtual world in which users have the perception of being and the ability to explore and interact with objects in it [3].

There are three different types of VR, first person VR or immersive VR where users totally immersed in the virtual world. Projection semi-immersive VR using stereoscopic tables or Computer Automatic Virtual Environment (CAVEs), where the virtual environment is generated by a system of triple projection screens that create a VR booth. Second person semi-immersive VR where the users see themselves in the virtual world through a screen without losing contact with the real world. These semi-immersive VR models are generated by an image capture system or by a digital representation of the body or body part called avatar or virtual identity, which reproduces the movements of the person in the virtual environment. This type of VR often requires additional peripheral devices such as haptic gloves. Finally, desktop VR or non-immersive VR, which the person views the 3D virtual world on a monitor and interacts with the system through a controller, mouse, or joystick [3,4].

VR has a major role in promoting functional recovery poststroke. VR provides important benefits to the neuroplasticity process such goal-oriented task, real-time feedback, altering task difficult and increased users’ motivation and enjoyment [5]. Some of these benefits are related to the motor planning process which may contribute to the UE motor function improvement [6,7]. Furthermore, VR presents the possibility to implement effective intervention with some systems designed at low cost, which has reduced one of the main limitations of these systems described in the literature [8].

Exoskeletons are electro mechanized robots whose joints must match those of the patient, allowing greater control over movement [9]. On another note, haptic systems, mainly gloves, provide greater interaction between the user and virtual objects. Moreover, these systems facilitate the integration of visual information and neuromuscular feedback, allowing to rehabilitate manipulation tasks in an effective way. Haptic proprioceptive information and visual feedback must be synchronized in time and space [10].

The combination of exoskeletal/glove support and VR was supposedly able to further promote the UE function recovery in stroke patients. Since a large number of repetitions or high intensity rehabilitation can be performed with the help of these devices [8]. Due to the potential of this combination, multiple gloves have been developed with the intention of facilitating and improving UE rehabilitation after stroke [11]. Given the need for new systems to improve motor recovery after stroke, haptic gloves are presented as one of the systems that provide greater feedback and interaction between the user and virtual objects; since haptic gloves in rehabilitation are mostly combined with semi-immersive VR (SVR), a review of the combined use of these systems in the in the short (less than 1 month post-rehabilitation) and long term (more than 1 month post-rehabilitation) UE motor rehabilitation in people with stroke is needed.

## 2. Materials and Methods

### 2.1. Design

This meta-analysis and systematic review were conducted following the Preferred Reporting Items for Systematic Reviews and Meta-Analyses (PRISMA) recommendations for the communication of systematic reviews [12].

Systematic reviews are essential for healthcare providers and decision makers due to the huge volume of research on which to base their decisions. To allow for these decisions and to understand their possibility to replicate the review findings, PRISMA was designed to help researchers to prepare transparent accounts of their reviews, and its recommendations have been widely endorsed and adopted.

The protocol of this systematic review and meta-analysis was registered prior to its publication in PROSPERO with the registration number: CRD42022339962

### 2.2. Search Strategy and Database

In May 2022, the following databases were consulted, answering the research question in PICO format: PubMed, CINHAAL COMPLETE, The Web of Science, PEDro, SCOPUS and The Cochrane Database. Keywords referring to the pathology, the UE, and the intervention were used, combined with Boolean operators (complete search strategy is showed in Appendix A Table A1).

### 2.3. Screening Process and Eligibility Criteria

Study titles and abstracts obtained from the databases were analyzed by two different investigators according to the established inclusion criteria and discrepancies were resolved by a third investigator. 

The inclusion criteria included: randomized clinical trials in English, Spanish, Portuguese and French that performed an intervention using rehabilitation gloves combined with SVR in people who had suffered a stroke. To be considered eligible, studies had to present: persons who had suffered a stroke at any stage of the disease; assess UE functionality using standardized scales; and had a mild spasticity (defined as modified Ashworth scale score of <3 in any of the shoulder, elbow, or wrist/finder muscles).

The exclusion criteria included: other neurological impairments; the variables of interest were not reported; diseases other than stroke; non-semi-immersive VR; no combination of rehabilitation gloves with SVR.

### 2.4. Data Extraction

A standardized methodology was used to obtain data from studies that met the criteria. Data were obtained on the first author, year of publication, design, number of patients, patient demographics, type of device used in rehabilitation, treatment characteristics, and study outcomes (UE functionality). For post-treatment assessment, less than 1 month was considered short-term, and more than 1 month was considered long-term. In addition, the means and standard deviations of the study results were obtained. The authors of the included studies were contacted by e-mail, with the aim of accessing possible unclear data. If no response was received, the data were excluded from the analysis.

### 2.5. Assessment Methodological Quiality of the Studies and Risk of Bias

To analyze the methodological quality of each study, the Physiotherapy Evidence Database (PEDro) was used [13]. This scale includes 11 items, with the maximum score being 10, since the first item is not used to calculate the total score, but studies that do not meet this item should be excluded. Scores of 9 and 10 indicate that the studies are of excellent quality, 6 to 8 of good quality, 4 to 5 of fair quality, and <4 of poor methodological quality. 

To assess the risk of bias of the included studies, we used the modified Cochrane library criteria [14]. This scale scores the risk of bias of the studies according to different conditions such as “high”, “low” or “some concern”. Discrepancies were resolved by a third investigator throughout the process of analyzing methodological quality and risk of bias.

Additionally, the articles were classified according to the levels of evidence and grades of recommendation for diagnosis studies established by Oxford Center for Evidence-Based Medicine [15].

### 2.6. Data Synthesis and Analysis

The quantitative analysis included studies comparing the differences between stroke individuals treated with rehabilitation gloves and SVR and stroke subjects treated with conventional therapy. The analysis was performed on variables related to UE functionality. The mean differences (MD) between pre-intervention and post-intervention were used to detect the comparison values between groups. The mean difference between groups was used when measurements were collected in the same unit and with comparable assessments; means were converted to the standardized mean difference (SMD), with a 95% confidence interval (CI) to obtain the effect size, or when means were not comparable. A random-effects model was used to determine the overall effect size: in the SMD, an effect size of >0.8 was considered large, between 0.5 and 0.8 was considered medium, and between 0.2 and 0.5 was considered small [16], and *p* values < 0.05 were considered statistically significant. The degree of heterogeneity between studies was estimated using Cochran’s Q statistical test (with *p* values < 0.05 considered significant) [17] and the inconsistency index (I^2^). An I^2^ > 25% was considered to represent small heterogeneity, an I^2^ > 50% medium, and an I^2^ > 75% large. The I^2^ is a complement to the Q test, although it has the same power problems when the number of studies is small [17]. When the Q test was significant (*p* < 0.1) and/or the I^2^ score was >25%, indicating heterogeneity among studies, the random-effects model was applied in the meta-analysis. Asymmetry was assessed using a funnel plot in those analyses consisting of at least five studies, indicating the possible risk of publication of small studies with negative results. The studies were analyzed with Review Manager 5.4 Review Manager (RevMan; Computer program, The Cochrane Collaboration, 2020) statistical software.

## 3. Results

### 3.1. Studies Selection 

**Figure 1:**
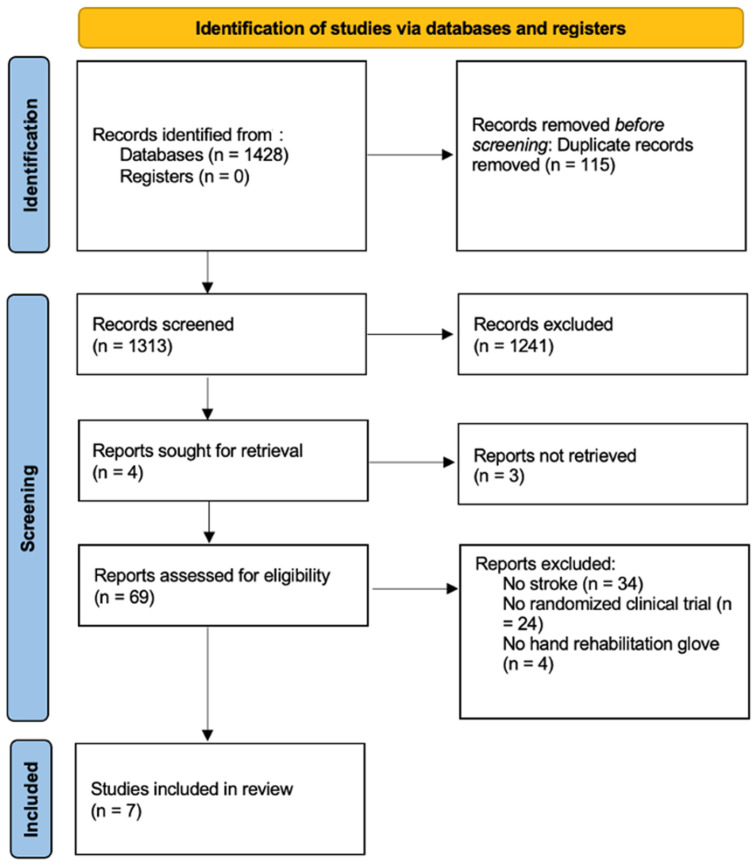
PRISMA Flowchart diagram.

A total of 1428 studies were retrieved. Duplicate studies were eliminated, leaving a total of 1313 studies, on which a critical reading of the title and abstract was carried out. After first screening, there was a total of 71 studies, which were obtained and read in full text. Finally, 7 studies [18,19,20,21,22,23,24] were included in the qualitative analysis, and quantitative analysis, with a total of 230 subjects. The screening process is shown in the PRISMA flow diagram (Figure 1).

### 3.2. Characteristics of the Included Studies

The age range of the 230 patients was between 42 and 78 years, all studies included hemorrhagic and ischemic stroke. Kang et al. [18], Park et al. [20] and Patel et al. [21], conducted the studies with acute post-stroke patients (<1 month), while the rest of the studies recruited chronic post-stroke patients (>12 months). The number of sessions ranged from 8 to 24, with a duration between 30 min to 1 h and with a minimum intensity of 3 sessions per week. All of this data and the device used in each study is in Table 1. No adverse events or cybersickness were reported in any of the studies.

### 3.3. Outcome Measures

Different methods were used to assess UE functionality in the included studies. Of the included studies, five used the Fugl Meyer UE scale (FMA-UE) [17,19,20,22,23], five used the Jebsen-Taylor hand function test (JTT) [18,19,20,23,24], three used the Block and Box test (BBT) [18,19,22], three analyzed the hand grip strength (HGS) [18,20,24], one used the Wolf Motor Function Test (WMFT) [19], one used the action research arm test (ARAT) [22], and one used the Chedoke-McMaster Armand Hand Activity Inventory [24]. Seven studies evaluated the UE functionality in the short-term [18,19,20,21,22,23,24], and five evaluated the UE functionality in the long-term [18,21,22,23,24].

### 3.4. Quality Assessment

**Figure 2:**
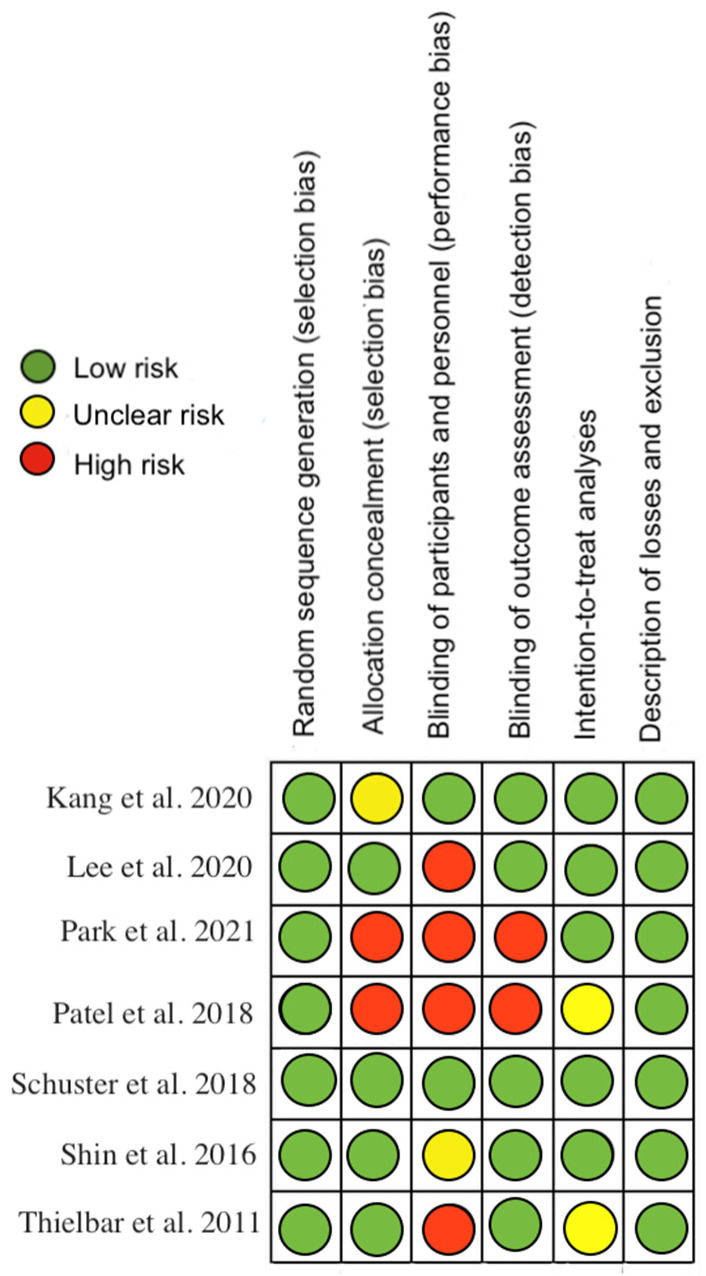
Risk of bias for each included study [18,19,20,21,22,23,24].

**Figure 3:**
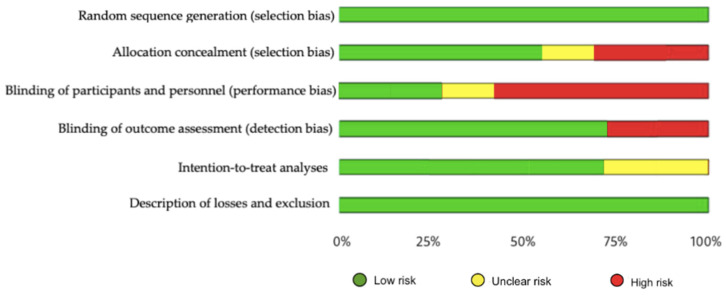
Risk of bias represented as percentages among all included studies.

The methodological quality, assessed by the PEDro scale, showed that one of the included studies had excellent methodological quality, five had good quality and 1 had fair quality (Appendix A Table A2). Regarding risk of bias, studies were at greatest risk in blinding of participants and staff, blinding of outcome assessment, and allocation concealment (Figure 2 and Figure 3). All articles were classified as level of evidence II, with a grade of recommendation of B (Appendix B Table A3).

### 3.5. Qualitative Summary of the Included Studies

#### 3.5.1. Short-Term Assessment of UE Functionality

**Figure 4:**
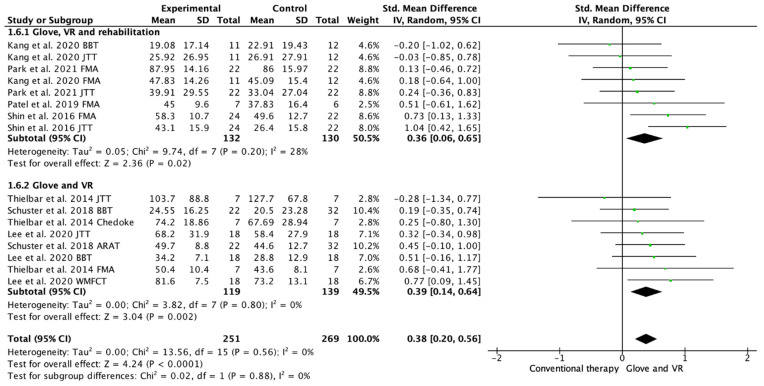
Forest plot of the results of a random-effects meta-analysis shown as standardized mean differences (SMD) with 95% confidence interval (CI) for short-term upper limb function assessment. Rehabilitation glove, combined with virtual reality and rehabilitation, and only rehabilitation glove, combined with virtual reality subgroups are reflected. The shaded square represents the point estimate for each individual study and the weight of the study in the meta-analysis. The diamond represents the overall mean difference of the studies [18,19,20,21,22,23,24].

Seven studies evaluated the UE functionality [18,19,20,21,22,23,24]. Among these studies, 16 outcome measures were included as some of the included studies assessed function with different scales. A total of 13 of the assessments showed improvements in favor of the experimental group [18,19,20,21,22,23,24]. Among the included studies, 4 performed a rehabilitation glove-based intervention combined with virtual reality and conventional rehabilitation after therapy [18,20,21,23], and 3 studies applied only rehabilitation glove-based rehabilitation combined with virtual reality [20,22,24]. The meta-analysis showed that the combined therapy of rehabilitation glove and SVR produced significant improvements in UE function compared to conventional therapy with a moderate effect size (SMD = 0.38, 95% CI = 0.20; 0.56; Z: 4.24; *p* =< 0.001, N = 520) (Figure 4). Low between-study heterogeneity was estimated (*p*: 0.56; I^2^: 0%). The funnel plot presents symmetry, indicating the low risk of publication bias (Appendix C). A subgroup analysis was performed according to whether they performed rehabilitation associated with the use of the rehabilitation glove combined with virtual reality or not (Figure 4). The subgroup analysis showed no significant differences between interventions (*p*: 0.88), showing a significant medium effect size in the use of rehabilitation associated with the use of the rehabilitation glove combined with virtual reality (SMD = 0.36, 95% CI = 0.06; 0.65; Z: 2.36; *p* = 0.002, N = 262) and in the use of rehabilitation glove combined with virtual reality without rehabilitation (SMD = 0.39; 95% CI = 0.14; 0.64; Z: 3.04; *p* =< 0.001, N = 258). There was homogeneity between subgroups (I^2^: 0%).

#### 3.5.2. Long-Term Assessment of UE Functionality

**Figure 5:**
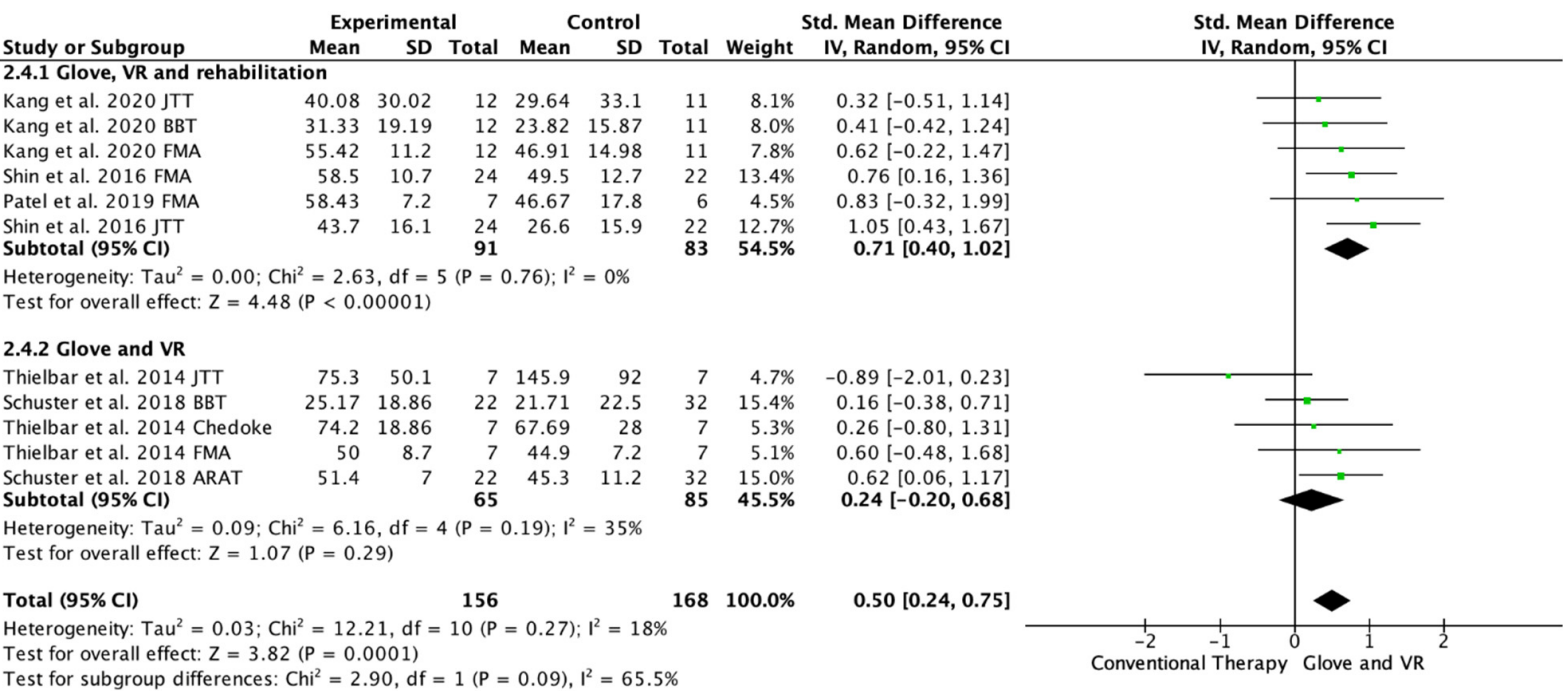
Forest plot of the results of a random-effects meta-analysis shown as standardized mean differences (SMD) with 95% confidence interval (CI) for long-term upper limb function assessment. Rehabilitation glove, combined with virtual reality and rehabilitation, and only rehabilitation glove, combined with virtual reality subgroups are reflected. The shaded square represents the point estimate for each individual study and the weight of the study in the meta-analysis. The diamond represents the overall mean difference of the studies [18,21,22,23,24].

Five studies evaluated the UE functionality in the long-term, from 1 month to 6 months of follow-up [20,21,22,23,24]. Among these studies, 11 outcome measures were included as some of the included studies assessed function with different scales; 10 of the assessments showed improvements in favor of the experimental group [20,21,22,23,24]. Among the included studies, three performed a rehabilitation glove-based intervention combined with virtual reality and conventional rehabilitation after therapy [18,21,23], and two studies applied only rehabilitation glove-based rehabilitation combined with virtual reality [22,24]. The meta-analysis showed that the combined therapy of rehabilitation glove and SVR produced significant improvements in the long-term UE function compared to conventional therapy in the long-term with a moderate effect size (SMD = 0.50, 95% CI = 0.24; 0.68; Z: 3.82; *p* =< 0.001, N = 324) (Figure 5). Low non-significant between-study heterogeneity was estimated (*p*: 0.27; I^2^: 18%). The funnel plot presents asymmetry, indicating the risk of publication bias (Appendix C). A subgroup analysis was performed according to whether they performed rehabilitation associated with the use of the rehabilitation glove combined with virtual reality or not (Figure 5). The subgroup analysis showed significant differences between interventions (*p*: 0.09) in favor of the use of rehabilitation associated with the use of the rehabilitation glove combined with virtual reality (SMD = 0.71, 95% CI = 0.40; 1.02; Z: 4.48; *p* =< 0.001, N = 174 vs. SMD = 0.24, 95% CI = −0.20; 0.68; Z: 1.07; *p* = 0.19, N = 150) (Figure 5). There was moderate homogeneity between subgroups (I^2^: 65.5%).

#### 3.5.3. Hand Grip Strength

**Figure 6:**
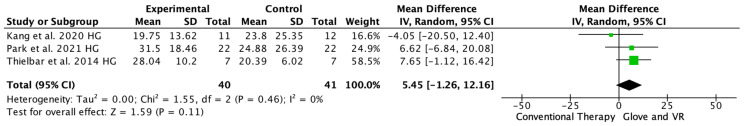
Forest plot of the results of a random effects meta-analysis on hand grip strength, shown as standardized mean differences (SMD) with 95% confidence interval (CI). The shaded square represents the point estimate for each individual study and the weight of the study in the meta-analysis. The diamond represents the overall mean difference of the studies [18,20,24].

Three studies evaluated the arm strength with the HGS [18,20,24], one study showed significant differences in favor of the VR and rehab glove group [20], and two showed no difference between the interventions [18,24]. The meta-analysis showed that the combined therapy of rehabilitation glove and SVR produced non-significant improvements in HGS compared to conventional therapy (MD = 5.45, 95% CI = −1.26; 12.16, *p* =< 0.11, N = 81) (Figure 6). Low between-study heterogeneity was estimated (*p*: 0.46; I^2^: 0%).

## 4. Discussion

The aim of this meta-analysis and systematic review was to analyze the short- and long-term effectiveness of the combined use of rehabilitation gloves with SVR in UE rehabilitation after stroke. Most studies included subjects with homogeneous characteristics in terms of demographic variables and baseline UE functionality. The available evidence seems to indicate that the combined use of rehabilitation gloves with SVR produces significant improvements over conventional rehabilitation treatment in the UE functionality of stroke patients. Both of the studies that used rehabilitation gloves combined with SVR, and studies that also performed conventional rehabilitation associated with gloves and SVR, were included in the present meta-analysis and review. The differences found with respect to short- and long-term effectiveness may lie in the application or not of rehabilitation combined with the use of gloves and SVR, since although in the short term, both types of study were superior to conventional treatment (Figure 4), in the long term, only those studies that performed rehabilitation in addition to rehabilitation with gloves and SVR, maintained significant improvements (Figure 5). The included studies showed no significant differences in hand grip strength.

### 4.1. Short-Term Upper Limb Functionality

The meta-analysis showed that the combined use of rehabilitation gloves with SVR produces significant improvements over conventional rehabilitation treatment in the UE functionality of stroke patients at short-term, regardless of whether or not associated conventional rehabilitation is performed. All or the included studies showed improvements supporting the combined use of rehabilitation gloves with SVR, so that between 8 and 24 sessions seem to be adequate for the motor rehabilitation of people with acute to chronic stroke, performing the treatment between 3 and 5 days per week, with a duration of each session of at least 30 min. Laver et al. [6] argued that was needed at least 15 h of RV rehabilitation to present better results, it is likely that the two studies included in this study with less than 15 sessions (Kang et al. [18] and Patel et al. [21]) have a similar improvement due to the intensity of sessions, performing 5 sessions per week on consecutive days.

The use of VR for upper extremity rehabilitation may have more effects than traditional approaches in UE motor rehabilitation following stroke, according to these results, which are in line with those of several writers [5,25].

The benefits obtained in conventional rehabilitation protocols that include VR may lie in the fact that VR would enhance rehabilitation improvement, as demonstrated by Singh et al. [26] in their study carried out in 2021, comparing a group of chronic stroke patients treated with VR and other group treated with conventional rehabilitation. Both groups showed improvements in functionality, but the VR group showed superior changes in cortical excitability than the control group [25]. Current meta-analysis [5] suggest that VR systems (specifically designed as well non-specific but adaptable) should introduce different elements in therapy to enhance clinical benefits. These elements include: variable activity-oriented practice, forcing use the affected hemibody, increasing difficulty and feedback systems.

### 4.2. Long-Term Upper Limb Functionality

The meta-analysis showed that the combined use of rehabilitation gloves with SVR produces significant long-term improvements (at least 1-month post-treatment) over conventional rehabilitation treatment in the UE functionality of stroke patients. Subgroup analysis showed that these changes are only preserved if, in addition to the use of rehabilitation gloves and SVR, conventional rehabilitation is applied. The studies that performed conventional rehabilitation associated with the use of the glove and SVR showed significant differences with respect to the group that did not perform associated conventional rehabilitation. The Cochrane Library in 2017 [6], through its latest systematic review on the use of VR (no rehabilitation gloves were included), focused on UE rehabilitation in people who have suffered a stroke, indicated that when VR is used as an adjunct to usual care (providing a higher dose of treatment for those in the intervention group) it provides significant improvements in the UE as long as 15 h of treatment is exceeded. However, it does not appear to achieve such results when comparing VR with conventional treatment in terms of UE function, so the differences may not lie in the associated use or not of rehabilitation, but in a higher dose of treatment. When these treatment doses are equivalent, no significant differences would be expected. Regarding the maintenance of long-term improvements, studies based on VR focused on recovery of the UE motor function after stroke have generally performed follow-ups between 1 and 6 months after treatment. Perhaps the difference could lie in the addition of a “haptic” component to the rehabilitation, as it could provide the patient with more feedback than a single VR treatment, being the difference between the two treatments. Nevertheless, the results found in the literature are contradictory, since there are studies that show that VR presents no differences with conventional therapy at 1 month follow-up after treatment [27,28] but there are authors who postulate that VR presents significant improvements in the function of the UE with respect to conventional therapy at 6-month follow-up [29].

Interestingly, in our meta-analysis and systematic review, the included studies that did not perform associated rehabilitation used the Bi-Manu Trainer [22] and PneuGlove [24] systems, while studies that performed associated rehabilitation used the RAFAEL™ Smart Glove model except for one study [21]. The RAFAEL™ Smart Glove has flexible bending sensors in the finger parts and inertial measurement unit sensors in the wrist, both sensors compute the amount of individual finger movement and the position and movement of the hand and wrist. The application provides and accurately visual feedback of finger, wrist, and hand in real time. To keep patients engaged and to make the exercises steadily more difficult, this system included a learning schedule algorithm to game-like exercises. The learning schedule algorithm proposes an ideal difficult task to improve learning of numerous functional activities. The specific characteristics of this system could explain the differences found with respect to follow-up times.

### 4.3. Hand Grip Strength

Our meta-analysis showed that the combined use of gloves with SVR in rehabilitation for stroke patients do not significantly improve the hand grip strength over conventional rehabilitation, which seems similar to the findings of a case of series that performed similar interventions to the included in this meta-analysis [30].

The combination of gloves and SVR with conventional rehabilitation has no greater effect that conventional rehabilitation alone. So, it seems that VR does not achieve strength gains superior to conventional rehabilitation.

Currently, low-cost, and commercial systems have a great relevance in the rehabilitation treatment of stroke patients with motor symptomatology. For example, according to the literature, the Kinect + XBOX^®^ system, in combination with conventional rehabilitative treatment, appears to be superior to conventional treatment in terms of motor function, range of motion and upper limb coordination in stroke patients [31]. Nevertheless, these systems, because of their ease of use, have the disadvantage of having a great heterogeneity of the protocols used; the Kinect + XBOX^®^ system is usually recommended in 12–40 sessions, 2–5 times/week, 30–120 min per session. The four most popular commercial programs are typically: Kinect Sports I and II^®^, Kinect Adventures^®^ and Kinect Gunstringer^®^ [32].

With regards to the strengths of this paper, this systematic and meta-analyses was conducted following PRISMA recommendations. Moreover, the protocol was registered prior to its publication in PROSPERO. For the systematic review, the methodological quality of each included study was assessed by PEDro. In addition, the level of evidence and grade of recommendation of each paper was explored. Finally, risk of bias of the included studies was studied by the modified Cochrane library criteria. Therefore, we consider it to be a fully replicable systematic review and meta-analysis.

There are clinical implications derived from this systematic review and meta-analysis to be considered. The recommendations of using haptic gloves system in a rehabilitation context, combined with semi-inversive VR, according to this review and previous bibliography, are based on a minimum of 15 treatment sessions (10 is the minimum number included in the protocols), applied intensively (3–5 sessions per week), with a duration of the sessions between 30 min and 1 h of virtual reality. It is also advisable to associate conventional rehabilitation, since in this way, the effects could be enhanced, and the improvements in the upper limb could be maintained in the long term of 1 to 6 months. This protocol could be used by patients with acute, sub-acute and chronic stroke. The most used device in the literature is the RAFAEL™ Smart Glove system, although low-cost systems may be used with similar clinical effects based on the results of our meta-analysis. Finally, it is noteworthy that no adverse events or cybersickness were reported in any of the studies included in this systematic review and meta-analysis.

Future studies are needed with higher methodological quality, including a representative number of patients, clearly categorizing their clinical symptomatology, and assessing upper limb functionality using standardized scales, a such as the FMA, and with follow-up times.

### 4.4. Limitations

The present study showed some limitations. We cannot extrapolate our results to all patients with stroke and at all stages of disease progression since the inclusion and exclusion criteria of the patients in the studies analyzed must be taken into account. Different outcome measures of upper limb functionality were included, since the included studies assessed UE functionality using different tools; the studies had different follow-up times; only 3 studies included in the grip strength variable as an outcome. The laterality of the subjects was not analyzed since most of the included studies did not provide this information. The included studies involved stroke patients with a spasticity of 3 points according to the Ashworth scale, but also studies that included patients with a spasticity below 2 points according to this same scale, which could influence the results. Although most of the included studies were of good methodological quality, in terms of risk of bias, the studies were at risk of blinding of participants, blinding of outcome assessment, and allocation concealment; therefore, the results should be read with caution.

## 5. Conclusions

The combined use of rehabilitation gloves with SVR produces significant improvements over conventional rehabilitation treatment in the UE functionality of stroke patients. If, in addition to the use of rehabilitation gloves and SVR, conventional rehabilitation is applied, improvements in UE are maintained at least one month after treatment. Recommended treatment protocols include a minimum of 15 sessions, applied intensively (3–5 sessions per week), performing VR for 30 min and 1 h.

## Figures and Tables

**Table 1:** Subjects and intervention characteristics.

Study	Population (Number, Men, Age)	Time Since Stroke	Devices	Therapy	Number of Sessions	Outcome Resources	Follow-Up
2020 Kang et al. [18]	23 (12, 57.78 ± 15.25)	24.87 ± 14.97 days	RAFAEL™ Smart Glove	30 min VR + 30 min CT	10 sessions, 5 per week	FMA-UE, HGS	1 month
2020 Lee et al. [19]	36 (27, 72.65 ± 5.55)	15.15 ± 15 months	RAFAEL™ Smart Glove	30 min VR	24 sessions, 3 per week	JJT, BBT, WMFT	-
2021 Park et al. [20]	44 (24, 61.44 ± 16.05)	≤1 month	RAFAEL™ Smart Glove	30 min VR + 30 min CT	20 sessions, 5 per week	FMA-UE, JJT, HGS	-
2019 Patel et al. [21]	13 (9, 59.31 ± 10.89)	12.5 ± 8.8 days	NJIT-RAVR system and CyberGlove Cybergrasp	1 h VR + 2 h CT	8 sessions, 5 per week	FMA-UE, WMFT	6 months
2018 Schuster et al. [22]	54 (39, 61.2 ± 12.3)	36 ± 60.6 months	Bi-Manu Trainer	45 min VR	16 sessions, 4 per week	BBT, CAHAI-13	2 months
2016 Shin et al. [23]	46 (36, 57.2 ± 10.3)	13.6 ± 13.4 months	RAFAEL™ Smart Glove	30 min VR + 30 min CT	20 sessions, 5 per week	FMA-UE, JTT	1 month
2014. Thielbar et al. [24]	16 (9 56.93 ± 7.1)	46.6 ± 32.5 months	PneuGlove	1 h VR	18 sessions, 3 per week	FMA-UE, JTT, HGS, ARAT	1 month

ARAT: Action Research Arm Test; BBT: Box & Block Test; CAHAI-13: Chedoke McMaster Arm and Hand Activity Inventory; CT: Conventional Therapy; FMA-UE: Fugl-Meyer assessment scale upper extremity; HGS: Hand grip strength; JTT: Jebsen-Taylor hand function test; VR: Virtual Reality; WMFT: Wolf Motor Function Test.

**Figure A1:**
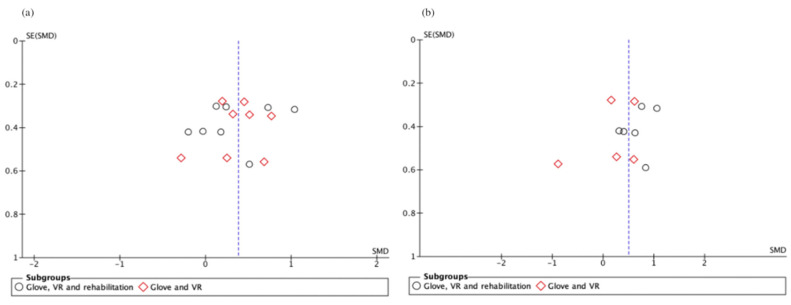
Funnel plot of short-term (**a**) and long-term (**b**) upper limb functionality studies. Slight asymmetry, meaning possible publication bias.

## Data Availability

All the papers included in this systematic review have been downloaded from the original publisher sites and are available to any researcher. Some of the publisher platforms may require a subscription to be able to read and download the papers.

## Appendix A

**Table A1:** Complete search strategy.

**Pathology Terms**	**Upper Limb Terms**
- “Stroke” - “Cerebrovascular accident” - “CVA”	- “Hand” - “Fingers” - “Finger” - “Upper extremity”
**Rehabilitation Glove Terms:**	**Virtual Reality Terms**
- “Exoskeleton Device” - “Exoskeleton device” - “Exoskeleton” - “Glove”	- “Virtual reality” - “Virtual reality exposure therapy” - “Virtual reality exposure therapy” - “Video games” - “Videogames” - “Virtual games” - “Augmented reality”
**Rehabilitation Terms**	
- “Rehabilitation” - “Physical and Rehabilitation Medicine” - “Neurological Rehabilitation” - “Rehabilitation” - “Physical and Rehabilitation Medicine”	

**Pathology terms:**

“Stroke” OR “Cerebrovascular accident” OR “CVA”

**Upper Extremity terms:**

“Hand” OR “Fingers” OR “Finger” OR “Upper extremity”

**Rehabilitation Glove terms:**

“Exoskeleton Device” OR “exoskeleton device” OR “Exoskeleton” OR “Glove”

**Virtual Reality terms:**

“Virtual reality” OR “virtual reality” OR “virtual reality exposure therapy” OR “virtual reality exposure therapy” OR “video games” OR “video games” OR “videogames” OR “virtual games” OR “Augmented reality” OR “Augmented reality”

**Rehabilitation terms:**

“Rehabilitation” OR “Physical and Rehabilitation Medicine” OR “Neurological Rehabilitation” OR “Rehabilitation” OR “Physical and Rehabilitation Medicine” OR “Neurological Rehabilitation”

(“virtual reality” OR “virtual reality”[Mesh] OR “virtual reality exposure therapy” OR “virtual reality exposure therapy”[Mesh] OR “video games”[Mesh] OR “video games” OR “videogames” OR “virtual games” OR “Augmented reality” OR “Augmented reality”[Mesh]) AND (“Rehabilitation”[Mesh] OR “Physical and Rehabilitation Medicine”[Mesh] OR “Neurological Rehabilitation”[Mesh] OR “Rehabilitation” OR “Physical and Rehabilitation Medicine” OR “Neurological Rehabilitation”) AND (“Hand”[Mesh] OR “Fingers”[Mesh] OR “Finger” OR “Upper extremity”) AND (“Exoskeleton Device”[Mesh] OR “exoskeleton device” OR “Exoskeleton” OR “Glove”)

**Table A2:** Methodological quality assessment of the included studies with PEDro scale.

	1	2	3	4	5	6	7	8	9	10	11	Total
Kang et al., 2020 [18]	Yes	1	0	1	0	1	1	1	0	1	1	7
Lee et al., 2020 [19]	Yes	1	1	1	0	0	1	1	1	1	1	8
Park et al., 2021 [20]	Yes	1	0	1	0	0	0	1	1	1	1	6
Patel et al., 2019 [21]	Yes	0	0	1	0	0	0	1	1	1	1	5
Schuster et al., 2018 [22]	Yes	1	1	1	0	1	1	1	1	1	1	9
Shin et al., 2016 [23]	Yes	1	1	1	0	0	1	1	1	1	1	8
Thielbar et al., 2011 [24]	Yes	1	0	1	0	0	1	1	1	1	1	8

1: Specified Study Eligibility; 2: Random Allocation of Subjects; 3: Concealed Allocation; 4: Similarity Between Groups at Baseline; 5: Subject Blinding; 6: Therapist Blinding; 7: Assessor Blinding; 8: <15% Dropouts; 9: Intention-to-Treat Analysis; 10: Between-Group Statistical Comparisons; 11: Point Measures and Variability Data.

## Appendix B

**Table A3:** Level of Evidence and Grade of Recommendation.

Study	Level of Evidence	Grade of Recommendation
Kang et al., 2020 [18]	**II**	**B**
Lee et al., 2020 [19]	**II**	**B**
Park et al., 2021 [20]	**II**	**B**
Patel et al., 2019 [21]	**II**	**B**
Schuster et al., 2018 [22]	**II**	**B**
Shin et al., 2016 [23]	**II**	**B**
Thielbar et al., 2011 [24]	**II**	**B**
